# Legumes Protease Inhibitors as Biopesticides and Their Defense Mechanisms against Biotic Factors

**DOI:** 10.3390/ijms21093322

**Published:** 2020-05-08

**Authors:** Lucio Rodríguez-Sifuentes, Jolanta Elzbieta Marszalek, Cristina Chuck-Hernández, Sergio O. Serna-Saldívar

**Affiliations:** 1 Facultad de Ciencias Biológicas, Universidad Autónoma de Coahuila, Carretera Torreón-Matamoros Km 7.5, Torreón Coahuila 27104, Mexico; lucio.rodriguez@uadec.edu.mx (L.R.-S.); j.marszalek@uadec.edu.mx (J.E.M.); 2 Tecnológico de Monterrey, School of Engineering and Sciences, Eugenio Garza Sada 2501, Col. Tecnológico, Monterrey Nuevo León 64849, Mexico; sserna@tec.mx

**Keywords:** antinutritional factors, enzyme activity inhibition, pathogenesis-related proteins, phytohormones, plant immune response, virulence factors

## Abstract

Legumes are affected by biotic factors such as insects, molds, bacteria, and viruses. These plants can produce many different molecules in response to the attack of phytopathogens. Protease inhibitors (PIs) are proteins produced by legumes that inhibit the protease activity of phytopathogens. PIs are known to reduce nutrient availability, which diminishes pathogen growth and can lead to the death of the pathogen. PIs are classified according to the specificity of the mechanistic activity of the proteolytic enzymes, with serine and cysteine protease inhibitors being studied the most. Previous investigations have reported the efficacy of these highly stable proteins against diverse biotic factors and the concomitant protective effects in crops, representing a possible replacement of toxic agrochemicals that harm the environment.

## 1. Introduction

Legumes (*Fabaceae*) are the second most important crops in the world, after the grass or Gramineae family (*Poaceae*) [1]. Legume seeds are widely used for direct food use because of their high nutritional content (Table 1) [2] and bioactive compounds (dietary fiber, polyphenols, flavonoids, phytosterols, resistant starch, and micronutrients like essential minerals and vitamins) [3,4,5].

The Table was modified from Kamboj et al. [2].

Like many other relevant crops, legumes are exposed to different environmental stresses that affect their yield. These stress factors can be abiotic or biotic. The latter include fungi, bacteria, viruses, nematodes, and herbivorous insects [6,7,8]. Legumes are especially attacked by different species of the *Fusarium* genus (*F. oxysporum, F. solani, F. udum,* and *F. virguliforme*). These molds are responsible for the formation of vascular wilt and root rot and, therefore, lead to reduced crop yield. Likewise, many phytopathogenic bacteria, such as *Pseudomonas* and *Xanthomonas,* negatively affect legume plants by causing leaf blights and spotting. Viruses are transmitted by insects and may cause different symptoms on different host plants. Viruses primarily predispose legumes to other pathogen infections [9]. The nematodes that are known to have the most significant impact on legumes are root-knot (*Meloidogyne* spp.) and cyst nematodes (*Heterodera* spp. and *Globodera* spp.); they cause substantial damage in legume crops [10]. The insects, *Callosobruchus chinesis,* and *C. maculatus,* are the most important pests of legumes because they damage the seeds, causing a loss of dry matter weight, nutritional quality, and germination or viability [11,12]. These biotic factors severely affect legume crops, which can lead to significant economic losses and reduced world food production.

Currently, the use of agrochemicals is the principal way to eliminate, control, or prevent the attack of biotic agents. However, due to their toxicity and danger to human health, there is a necessity to replace them with non-toxic or less toxic products.

Legume plants synthesize and accumulate molecules in response to biotic stressors, known as antinutritional factors (ANFs). ANFs are compounds that reduce the bioavailability of nutrients through the inhibition of enzymes involved in digestion or by chelating minerals during pathogen infestations. Importantly, some ANFs are known to have toxic effects in living organisms when consumed at high doses [5]. Despite the presence of ANFs, the use of legumes as human food sources is not limited by the presence of these compounds. Several effective methods are utilized to inactivate or reduce the activity of ANFs [13]. ANFs are classified as protein- and non-protein-based compounds. Several studies have shown their potential benefits, including their use as biopesticides, anti-cancer agents, weight control, immune-modulators, and hypocholesterolemia regulators; additionally, there are other essential health benefits [14,15].

In response to a pathogen attack, legumes produce protein-based ANFs called pathogenesis-related (PR) proteins. According to Van Loon [16], PR proteins are those proteins that are not or only at basal concentrations detectable in healthy tissues, but for which accumulation at the protein level has been demonstrated upon pathological conditions and related situations in at least two or more plant–pathogen combinations. Van Loon [17] also introduced the term “inducible defense-related proteins” for PR proteins.

PR proteins are classified into PR-1 through PR-17 and act against pathogens by different mechanisms such as cell wall degradation (glucanase, chitinase), oxidative activity (peroxidase, oxalate oxidase), protease inhibitor, protein degradation (endoprotease), membrane permeabilization (thaumatin-like, defensin, thionin, lipid-transfer protein), degradation of RNA (ribonuclease-like), and other unknown mechanisms [18]. PR proteins that act as protease inhibitors (PIs) are classified as PR-6. These proteins inhibit the activity of protease enzymes in the pathogens; therefore, they are unable to feed on the amino acids present in the plant. In the particular case of legumes, they are capable of producing a great variety of PIs. The natural defense mechanisms of these plants can be exploited to avoid or decrease the use of toxic agrochemicals. For this reason, in the present review, we discuss the potential use of these proteins as biopesticides to control biotic stresses in crops of economic importance.

## 2. Legume Responses to Pathogen Attack

**Figure 1:**
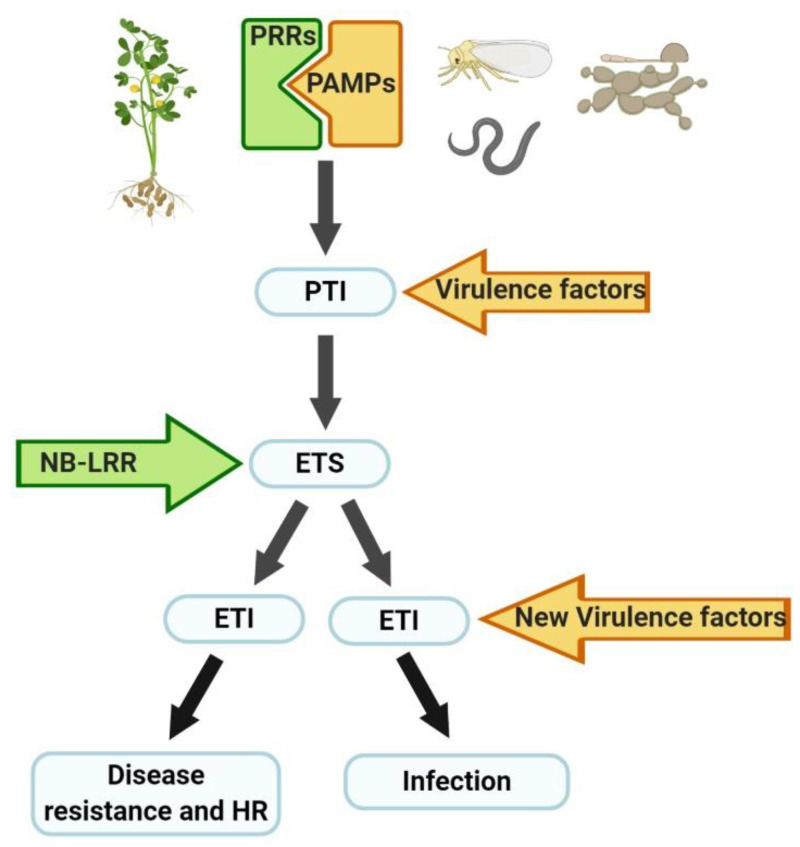
Plant defense mechanisms against the attack of pathogens. Pathogen recognition receptor (PRRs); pathogen-associated molecular patterns (PAMPs); PAMP-triggered immunity (PTI); effector-triggered susceptibility (ETS); nucleotide-binding and leucine-rich repeat domains (NB-LRR); effector-triggered immunity (ETI); hypersensitive cell death (HR).

Plants have developed different defense mechanisms in response to biotic stressors. As shown in Figure 1, when a pathogen is present, the plant uses cellular proteins, called pathogen recognition receptor (PRRs), to recognize inherent molecules of the pathogen, called pathogen-associated molecular patterns (PAMPs). PAMP-triggered immunity (PTI) is the result of this recognition process and is used by plants to initiate a response to cease or ameliorate pathogen colonization. Some pathogens are capable of producing effector molecules (virulence factors) that interfere with PTI, resulting in effector-triggered susceptibility (ETS). Plants can synthesize some NB-LRR (nucleotide-binding and leucine-rich repeat domains) proteins that recognize the pathogen effector, which in turn develops the effector-triggered immunity (ETI). ETI results in disease resistance and typically hypersensitive cell death (HR) at the infection site. Successful pathogens can avoid ETI by diversifying recognized effectors, or acquiring new effectors that eliminate NB-LRR proteins; this can lead to colonization infection [19,20,21].

**Figure 2:**
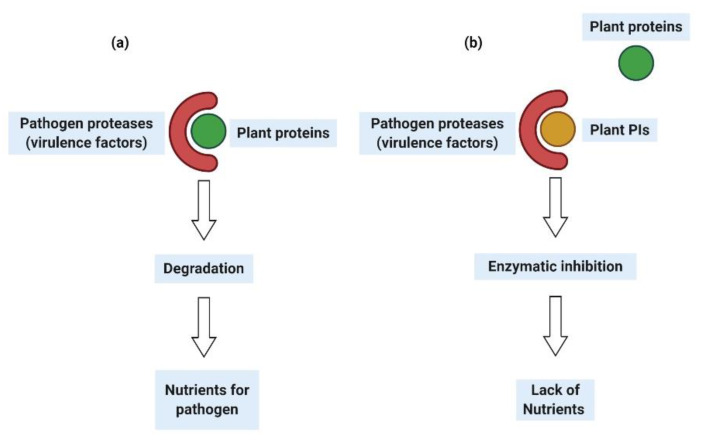
The action of pathogenic proteases (**a**), Action of protease inhibitors (PIs) over pathogen proteases (**b**).

Specific hydrolytic enzymes, also called virulence factors or pathogen effectors, are used by pathogens to degrade the plant cell wall to get nutrients from the plant. The ability of the pathogen to invade a plant is determined mainly by enzyme activity. The activity of pathogen enzymes, proteases, has been directly correlated to the severity of plant disease, which indicates their important role in pathogen colonization. The proteases destroy the plant cell wall structural proteins, allowing the pathogen to obtain nutrients for its growth, as well as to evade plant defenses [22,23] (Figure 2a). The inhibition of pathogenic enzymes by the plant PIs constitutes a plant defense mechanism. The main mechanistic action of PIs is the loss of the ability to colonize the host plant and nutrient deficiencies, which in turn causes a decrease in the growth or the reproduction capacity and possibly the death of the pathogen (Figure 2b). The ability of plants to produce active PIs in response to a pathogen attack is an important feature related to plant survival [24].

The mechanisms by which plants avoid pathogens attack draws great interest because understanding these mechanisms will help to find new strategies to control pathogens in crops. Since the legumes use PIs against pathogens, these proteins could be employed as useful biopesticide molecules in an environmentally friendly alternative to the toxic agrochemicals.

## 3. Phytohormones and PIs in Legumes

Some PR proteins are directed by the salicylic acid (SA)-dependent signaling pathway, while others are controlled by ethylene (ET) or jasmonic acid (JA). The phytohormones SA, ET, and JA act as both signaling or inhibiting molecules [25]. On the other hand, abscisic acid (ABA), produced in the presence of a pathogen, is a crucial phytohormone in the octadecanoid pathway that leads the production of JA and the consequent expression of PIs in tomato plants [26]. Phytohormones play an essential role in plant immunity against pathogens, and they can act in different ways in diverse plant parts according to the invasiveness of the pathogen. Phytohormones interactions involve complex crosstalk that has been reviewed by several authors [27,28,29].

In the case of legumes, defense responses are also elicited by phytohormones. It has been observed that soybeans accumulate SA after insect damage, which induces the expression of PIs-specific genes. Likewise, the external application of JA in soybean plants also induces PIs genes [30]. Additionally, it has been demonstrated that phosphatidic acid (PA) increases rapidly in wounded and neighboring unwounded soybean leaves, which activates the production of proteins related to plant defenses [31]. Paudel and Bede [32] indicated an increase in the activity of PIs, in the leaves of *Medicago truncatula,* under attack by beet armywood caterpillars (*Spodoptera exigua*). Additionally, in this same model, it was found that the JA and ET pathways were also activated. On the other hand, SA and ABA were not induced by insects.

In a transcriptomic study conducted by Wang et al. [33], a gene encoding PIs was induced in the leaves of two lines of soybean treated with the common cutworm (*Spodoptera litura fabricius*). Genes related to the JA signaling pathways were up-regulated under these conditions. Gao et al. [34] identified the *Acyrthosiphon kondoi* resistance gene, *AKR*, in response to a bluegreen aphid *A. kondoi* attack on *M. truncatula*. The bluegreen aphid *A. kondoi* infestation did not induce the genes involved in octadecanoic or JA pathways in a susceptible line of the plant. However, the PIs genes were induced by the aphid infestation in a resistance line. During aphid attack of susceptible and resistant lines, genes involved in the SA pathway were induced, and it was suggested that activation of this pathway might be a general mechanism of aphid repellence, with limited effectiveness in susceptible hosts. The octadecanoic acid pathway plays an essential role in the induction of PIs. Importantly, this metabolite participates in the synthesis of JA, which induces expression of PIs. *AKR*, a resistance gene (R-gene) that encodes to an NB-LRR protein, mediates JA defenses [34,35]. Yamchi et al. [36] confirmed the role of the JA pathway and the expression of PIs in the resistance of *M. truncatula* against the phytopathogenic bacteria, *Ralstonia solanacearum*.

Kunkel and Brooks [37] concluded that both the JA and ET are typically associated with defenses against insects and necrotrophic pathogens and that SA and JA/ET defense pathways are commonly mutually antagonistic.

There is no doubt that phytohormones play a crucial role in the defense mechanisms of legumes. According to recent studies, SA, JA, and ET seem to be the most relevant molecules that triggered the production of PIs in legumes. The ability of legumes to produce these phytohormones leads to pathogens attack and plant resistance.

## 4. PIs Present in Legumes

Plants carry resistance genes (*R*-genes), which are expressed to produce PR proteins under physical (wound signals), or chemical stimuli (biotic factors compounds). PR proteins are produced in diverse parts of the plants at basal concentrations and are overexpressed in the presence of pathogens, such as fungi, bacteria, viruses, nematodes, and insects. They represent a variety of weapons against pathogens since they possess hydrolytic activities, contact toxicity, involvement in defense signaling, and pathogen enzyme inhibition [17].

PR proteins are found in different molecular sizes ranging from 5 to 75 kDa and can be classified by family types, PR-1 through PR-17, according to their molecular weight, number of polypeptide chains, number of reactive sites, types of inhibition, and other features [16,18]. Importantly, the PR-6 family includes protease inhibitors. PIs act as natural plant defense proteins that form stable complexes with target pathogen proteases, thus blocking or preventing access to the enzyme’s active site [26,38]. According to the action mechanism of proteolytic enzymes and depending on the active amino acid in their active site, PIs can be divided into serine, cysteine, aspartic, and metalloprotease inhibitors [39]. Serine PIs are competitive inhibitors and prevent the activity of serine proteases such as trypsin, chymotrypsin, and elastase. These PIs are not used by plants to regulate endogenous protease activity. Nonetheless, they block protease activity in conditions that would be harmful to the plant [40]. Serine PIs are the most studied and are widely present in legumes, where two types have been identified, Kunitz and Bowman-Birk. Kunitz type has one or two polypeptide chains of 20 kDa, with four cysteine residues that form two intrachain disulfide bonds and inhibit trypsin activity. The Bowman-Birk PIs are single-chain polypeptides of 8 KDa, cysteine-rich with seven disulfide bonds, and two active domains for trypsin or chymotrypsin inhibition [15,40].

Cysteine PIs are the second most studied class of inhibitors, and they have a role in the regulation of endogenous and exogenous proteases. These proteins are competitive inhibitors that control several developmental processes involving cysteine proteases. They are capable of blocking papain-like cysteine proteases present in many insects and nematodes [26,39,41,42]. Cysteine PIs are grouped into the cystatin superfamily, which includes animal cystatins (stefins, cystatins, and kininogens) and plant cystatins, also called phytocystatins, which has more than 80 members. Phytocystatins are peptides ranging in size from 12–86 KDa; of note, some authors suggest that these kinds of inhibitors are the most promising PIs in transgenic plants [42].

Legumes react to pathogens attack by accumulating a large amount of PIs. Many plants can produce PIs; however, legumes are identified as the most important sources of these proteins. The PIs found in legumes have shown superior stability and superior inhibition activity over pathogens in comparison with PIs from other plants. Furthermore, legumes have a great variety of PIs, which means that these plants have developed expanded resistance against a wide range of pathogens.

Recently, a variety of studies have reported on the identification of PIs in many legumes, suggesting their potential as pesticides against biotic factors. Table 2 lists the most relevant PIs reported in recent studies. PIs represent a sustainable alternative to chemically contaminating products, and PIs have proven their effectiveness, principally against insects, nematodes, and fungi, as a greener alternative, compared to the use of chemical products that harm the environment.

## 5. Legume PIs as a Biopesticide against Insects and Nematodes

PIs influence the growth and development of insects and nematodes because they bind irreversibly to the active site of digestive gut proteases. Thus, these enzymes lose the ability to produce amino acids from the food, leading to the death of the organism [24].

Serine and cysteine proteases are the two essential enzymes present in the digestive system of insects. These proteases become the principal target of cysteine and serine PIs, produced by legumes, when under insect attack. Generally, serine PIs are effective against Lepidoptera and Diptera, whereas cysteine PIs are effective against Coleoptera. Both serine and cysteine PIs are active versus Aphids [39,46]. The effectivity of legume cysteine and serine PIs has been widely demonstrated in many crop insect pests.

*Helicoverpa armigera* is one of the most devastating insect pests in legumes. Swathi et al. [44] reported a potent inhibition of *H. armigera* gut trypsin-like protease (HGPs) by a serine PI present in pigeon pea (*Cajanus cajan*), a wild-type relative of *Cajanus platycarpus* (see Table 2). The inhibitor had high stability at 50 °C in acidic or basic conditions and contained several isoinhibitors that have also shown effectiveness against HGPs and exhibit high homology to Kunitz-type PIs. The authors further commented that the appearance of many isoinhibitors could be due to its wild nature, while domesticated varieties may have lost their genetic diversity [44]. Golla et al. [47] reported lower pod damage by *H. armigera* in wild relatives of chickpea (*Cicer arietinum*) compared to cultivated chickpea, due to the higher insect gut trypsin and chymotrypsin inhibition produced by PIs present in the wild-type plants.

Kaur et al. [46] observed a detrimental effect in the growth and development of *Batrocera cucurbitae*, a major pest of cucurbit crops when a soybean trypsin–chymotrypsin inhibitor was applied in vitro. In this study, a decrease of 66% in pupation and 53% in emergence were reported. The authors demonstrated that the complete inhibition of insect trypsin activity by the Bowman-Birk inhibitor was the cause of the observed reduction in insect growth and development.

Ramalho et al. [50] purified a PI from *Platypodium elegans* seeds, which resulted in a 98% and 30% inhibition in trypsin and chymotrypsin, respectively. This PI was stable at a temperature range from 30 to 80 °C and a pH range of 2 to 10. It also displayed a potent inhibition to trypsin enzymes obtained from the pest insects *Anagasta kuehniella, Corcyra cephalonica, Aedes aegypti,* and *Spodoptera frugiperda*. The larval weight of *S. frugiperda* decreased by 55%, and the extension of the insect life cycle increased, when feeding on the *P. elegans* PI.

The efficacy of cysteine PIs in pest insect elimination has also been reported. Studies carried out by Rondoni et al. [49] showed that oviposition of *Halyomorpha halys* nymphs on faba bean *(Vicia faba)* leaves increased gene expression of a cysteine PI in the plant, while the cysteine PI gene remained unchanged on plants whose leaves were not used for insect oviposition. An overexpression of JA-responsive *NAI1* and SA-responsive *PR1* genes was also observed on plants used for oviposition. The authors concluded that previous insect oviposition pre-activated plant defense systems, since lower weights of *H. halys* nymphs were observed. In comparison between the overexpression of trypsin and cysteine, *PI* genes indicated that only the second one was involved in defense mechanisms of faba bean against *H. halys*. Giacometti et al. [30] reported the efficacy of soybean cysteine PI against the invasive insect *Nezera viridula L* by diminishing the cysteine protease activity in the insect gut.

Nematodes are endoparasites that invade plant roots and therefore lead to considerable economic losses in agriculture. These losses are mainly due to two groups of nematodes; cyst (*Heterodera* and *Globodera* spp.) and root-knot (*Meloidogyne* spp.) [61]. Nematodes use gut proteases to assimilate proteins obtained from their host, an overexpression of *PI* genes in soybean roots, after infestation by *Heterodera glycines* has been observed as an essential defense mechanism [62].

Soybean seeds contain many PR proteins, of which PIs exert serine and cysteine PI activities. It is reported that cysteine proteases, in nematodes, play an essential role in protein degradation. Rocha et al. [51] observed the nematostatic and nematicidal effects of mature soybean seed exudate on *Meloidogyne incognita*. PR proteins, like chitinase, glucanase, and serine PIs, were identified in the exudate, and although the MS/MS analysis did not identify any phytocystatin, the exudate exerted inhibition against papain, a cysteine protease. The identification of a Kunitz type PI suggests its ability to inhibit serine and cysteine proteases, as mentioned by other authors [63,64].

In a study investigating *Crotalaria pallida*, Andrade et al. [52] identified a protein with a papain-inhibitory activity that did not belong to the family of PR-6 proteins. This PI protein was related to family PR-10 proteins, which have a single polypeptide chain of 15 KDa and exhibit a noncompetitive inhibition mechanism. In bioassays against *M. incognita,* the PR protein produced up to 95% mortality and was observed to internalize and diffuse over the entire body of the nematode.

Serine PIs in legumes may also contribute to plant resistance to nematode attack. Dawei et al. [53] studied the trypsin PI activity in two resistant and two susceptible soybean varieties to *H. glycines*. Inoculation of the nematodes on the plants increased trypsin PI activity in all the plants. Despite this, susceptible plants could not suppress invasion or spread of the nematodes in the root area, due to lower trypsin PI activity.

Serine and cysteine PIs show inhibition activity against insects and nematodes. More attention should be paid to cysteine PIs, since these inhibitors have been studied to a lesser extent and have shown high inhibitory effectiveness, especially against nematodes. Cysteine PIs are classified into a large group of proteins, within which are phytocystatins groups, more than 80 members of which are being studied for their use in transgenic crops.

## 6. Legume PIs as a Biopesticide against Phytopathogenic Fungi and Bacteria

Fungi can generate numerous diseases in plants and lead to devastating damage to crops. Phytopathogen fungi produce extracellular proteases that enter the plant cells and obtain nutrients. Plants have several mechanisms to defend against pathogenic fungi; one of these mechanisms is the production of PIs. Serine and cysteine PIs obstruct fungi pathogenic proteases and suppress the growth of the pathogen. However, the mechanism of antifungal action has not been fully elucidated [65].

Müller et al. [58] identified and characterized PIs produced by resistant and susceptible peanut cultivars to *Aspergillus spp*. Proteomic analysis showed the expression of three isoforms of Bowman-Birk type PIs in the two cultivars. A unique Bowman–Birk-type PIs isoform was found in the resistant cultivar, as well as a Kunitz-type PIs in response to *Aspergillus parasiticus* colonization. Extracellular fungi proteases caused a reduction of 82% in total activity when the PIs contained in the resistant cultivars were present. Antifungal activity was detected in the resistant cultivar due to the presence of Kunitz type PIs.

Wang et al. [59] showed the inhibition of the phytopathogenic fungi, including *Alternaria alternata, Fusarium oxysporum, Pythium aphanidermatum, Physalospora piricola, Botrytis cinerea,* and *Fusarium solani* by a PI from black soybean (*Glycine max L. merr.*). The PI inhibited trypsin and chymotrypsin activities, and its inhibitory activity was unaffected by temperatures up to 85 °C or within the pH range of 2–12. The isolation of the PIs involved ammonium sulfate precipitation and ion exchange and affinity chromatography. Amino acid sequences confirmed the high homology of this PI with the sequence of Bowman–Birk-type PIs. Wang et al. [60] also isolated a trypsin and chymotrypsin PI from mung bean (*Phaseolus mungo*), which was able to hinder the growth of phytopathogens fungi including *Physalospora piricola, Mycosphaerella arachidicola, Botrytis cinerea, Pythium aphanidermatum, Sclerotium rolfsii,* and *Fusarium oxysporum.*

Nair and Sandhu [56] observed a 99% reduction in trypsin activity with the presence of a PI in chickpea (*Cicer arietinum*)-resistant cultivar to the phytopathogenic fungus *Fusarium oxysporum*. Likewise, the growth of *F. oxysporum* decreased by 67% in the presence of crude extract from the legume, whereas a fraction of crude extract containing the trypsin PI reduced the conidial germination by 45% to 82%.

Cysteine PIs may contribute to the inhibition of phytopathogenic fungi growth since these PIs are also present in legumes. Scarafoni et al. [57] identified chitinase and glucanases (both PR proteins) and three isoforms of cystatins in the exudate of mature lupin seeds (*Lupinus albus*). This exudate inhibited the growth of the phytopathogens *Fusarium oxysporum, Botrytis cinerea, Alternaria solani, Aspergillus niger,* and *Penicillium expansum, by* approximately 15%, 50%, 42%, 80%, and 78%, respectively.

The authors of this review were not aware of studies relating to the effect of legume PIs on the growth of phytopathogenic bacteria. Nevertheless, Kim et al. [66] reported that Potide-G, a 5.5 KDa peptide from potato tubers, suppressed the proteolytic activity of trypsin, chymotrypsin, and papain and inhibited the growth of *Clavibacter michiganensis* subsp. *michiganensis*, an important pathogen of tomato plants. On the other hand, PIs from legumes, like *Inga vera,* has been reported to have bacteriostatic activity against *Escherichia coli* [67] or *Hymenaea courbaril,* with inhibitory activity against the pathogenic bacteria, *Vibrio parahaemolyticus*, *Staphylococcus aureus*, and *E. coli* [68]. The bacteriostatic effects of PIs from *Cajanus cajan* and *Phaseolus limensis* on gram-positive and harmful human pathogenic bacteria was reported by Shamsi et al. [69]. These reports indicate the enormous potential of legume PIs against phytopathogenic bacteria and the importance of conducting further studies in this area.

Unlike a large number of studies on legume PIs against insects, few studies have focused on the use of these inhibitors against the phytopathogenic fungi and bacteria. Because these proteins have shown good potential against this type of microorganisms, more research is needed in this area. This research would bring a more profound comprehension of these processes that represent an excellent opportunity for further applications since crops are affected by these pathogens around the world.

## 7. Recombinant PIs for Biotechnological Application

The application of PIs in agriculture can be implemented through two main strategies: (1) production of transgenic plants with the incorporation of *PIs* genes into their genomes or (2) production of recombinant PIs by microorganisms for use as biopesticides.

Several studies have reported a higher accumulation of ANFs like PIs, allergenic compounds, and lower protein quality in genetically modified crops [70,71]. Additionally, in the strategy of implementing PIs through transgenic plants, the phytopathogens can develop resistance against inhibitors intrinsically produced by the plant. Therefore, it would be pertinent to generate cultivars containing new *PIs* genes. Importantly, insects can adapt to the presence of PIs by several mechanisms, such as increasing digestive enzyme activity, decreasing the production of inhibitor-sensitive enzymes, digesting PIs, decreasing the sensitivity of proteases to PIs, and synthesizing more enzymes that are PI-resistant. Some pathogenic bacteria effectors can interfere with the plant defense system by suppressing the production of JA.

Additionally, it has been shown that endosymbiotic insect bacteria can produce non-sensitive PI proteases, which helps insects to digest protein in their gut [72,73,74]. Zhu-Salzman and Zeng [24] developed a wide range of transgenic plants carrying various types of foreign *PIs* genes. However, no PI-transgenic plants are commercially available because pathogen resistance against PIs has been detected.

Wild-type relatives of common legumes contain a pool of active genes against diverse pathogens, which make them resistant to diseases [75]. These genes have been used in the production of recombinant PIs in microorganisms. Using wild-type genes may prove to be a better strategy because the simultaneous application of different PIs in the crop would be possible. This strategy assures PI effectiveness by avoiding phytopathogen adaptation. This strategy has recently been utilized with bacteria and yeasts.

Mohanraj et al. [76] selected a PI obtained from *Rhynchosia sublobata*, a wild-type relative of pigeon pea. The gene of this PI was expressed in *Escherichia coli,* and the recombinant protein produced demonstrated inhibitory activity against trypsin, chymotrypsin, and gut trypsin-like protease activity of *A. janata* and *H. armigera*. Moreover, the recombinant PI was stable at 100 °C and a pH range of 2–12. In vivo feeding experiments showed retardation in growth and a mortality of up to 61% in *A. janata*. Monteiro Júnior et al. [77] produced two recombinant phytocystatins from cowpea (*Vigna unguiculata*) in *E. coli*. The proteins experienced high stability, potent inhibition of papain and chymopapain, and a decrease, up to 80%, in the total proteolytic activity of midgut proteases from *C. maculatus*. Contradictorily, the percentages of larval mortality, adult emergence, the average weight of emerged adults, and mean development time of *C. maculatus,* were not affected by any of the recombinant PIs. The authors argued that the beetle adapted to the diet and, as a result, produced cysteine protease-resistant to phytocystatins [78] and proteases capable of degrading the inhibitor [79]. On the other hand, Cruz et al. [11] reported the resistance to infestation and damage by *C. maculatus* in different varieties of Brazilian cowpea cultivars.

The yeast *Pichia pastoris* has been commonly employed in the production of recombinant proteins, including PIs, because of its high growth rate, ease of cultivation, and the ability to produce heterologous proteins. Luo et al. [80] expressed a PI from Malaytea scurfpea fruit (*Psoralea corylifolia* L.) in *P. pastoris*. The recombinant protein inhibited mycelium growth in important phytopathogenic fungi, such as *Aspergillus niger*, *Rhizoctonia solani,* and *Alternaria brassicae* and reduced conidial germination of *Alternaria alternate* by 50%.

Direct extraction of PIs from legume seeds is a challenging and impractical task. It can also result in an ethical problem due to the vast waste of food. On the other hand, the development of transgenic plants or the production of recombinant proteins represents practical strategies. The production of a biopesticide containing several PIs can also be advantageous, so the use of recombinant microorganisms for such preparation could be a great alternative.

## 8. Conclusions

Phytopathogenic insects, nematodes, fungi, and bacteria produce proteolytic enzymes to infect, colonize, and obtain nutrients from legumes. Legumes contain a broad set of weapons to counteract attacks by phytopathogens. Among these, PIs are natural proteins expressed in the presence of phytopathogens and are capable of inhibiting the activity of proteolytic enzymes. The efficacy of PIs to hinder or control phytopathogens has been extensively described.

For this reason, their use in transgenic plants or biopesticides is being explored. The best alternative to exploit PIs in agriculture is to produce them in recombinant microorganisms. This option represents the possibility of applying different PIs simultaneously in crops to avoid the development of phytopathogen resistance. An essential point in the use of PIs in agriculture is to select PIs with high stability, assuring their activity in the field. Additionally, the exploration of wild-type legume plants as PIs sources represents a good alternative in producing inhibitors with the potential to be used as co-adjutants in crop production.

## Figures and Tables

**Table 1:** Nutritional composition of different legume seeds (values in percentages).

Legume Seed	Protein	Fat	Minerals	Crude Fiber	Carbohydrates
Chickpea (*Cicer arietinum*)	17.1	5.3	3.0	3.9	60.9
Soybean (*Glycine max*)	43.2	19.5	19.5	3.7	20.9
Lentil (*Lens esculenta*)	25.1	0.7	2.1	0.7	59.0
Cowpea (*Vigna catjang*)	24.1	1.0	3.2	3.8	54.5
Peas dry (*Pisum sativum*)	19.7	1.1	2.2	4.5	56.5
Pigeon pea (*Cajanus cajan*)	22.3	1.7	3.5	1.5	57.6
Kidney bean (*Phaseolus vulgaris*)	22.9	1.3	3.2	4.8	60.6

**Table 2:** Legumes protease inhibitors (PIs) reported effective against phytopathogenic insects, nematodes, and fungi.

Seed Legumes	Protease Target	Insect Pest Target	Molecular Mass (kDa)	N-terminal Amino Acid Sequence	Reference
Soybean (*Glycine max*)	Trypsin and chymotrypsin	*Spodoptera littoralis*	17.9	ND	[43]
Pigeonpea (*Cajanus cajan, Cajanus platycarpus*)	Trypsin and chymotrypsin	*Helicoverpa armigera*	ND	ND	[44]
Soybean (*Glycine max*)	Trypsin	*Spodoptera litura*	ND	ND	[45]
Soybean (*Glycine max*)	Trypsin	*B. cucurbitae*	ND	ND	[46]
Chickpea (*Cicer arietinum, Cicer cuneatum, Cicer bijugum, Cicer chrossanicum, Cicer reticulatum*)	Trypsin and chymotrypsin	*Helicoverpa armigera*	ND	ND	[47]
Common bean *(Phaseolus vulgaris*)	Trypsin	*Spodoptera litura*	ND	ND	[48]
Faba bean *(Vicia faba)*	Cysteine protease	*Halyomorpha halys*	ND	ND	[49]
Uruvalheira (*Platypodium elegans)*	Trypsin and chymotrypsin	*Spodoptera frugiperda*	19.7	FVVDTDGDPLRNGGSYYILPVFRGRGGGIEQAAIGTETCPLTVVQSPSEVSKGLPLR	[50]
Soybean (*Glycine max*)	Cysteine protease	*Nezara viridula L.*	ND	ND	[30]
**Seed Legume**	**Protease Target**	**Nematode Pest Target**	**Molecular Mass (kDa)**	**N-terminal Amino Acid Sequence**	**Reference**
Soybean (*Glycine max)*	Trypsin and papain	*Meloidogyne incognita*	4.53–22.546	ND	[51]
*Crotalaria pallida*	Papain	*Meloidogyne incognita*	15	FAFEDENTSPVAPAKLFKALTKDADVIIPKVIEPDQ	[52]
Soybean (*Glycine max*)	Trypsin	*Heterodera glycines*	ND	ND	[53]
**Seed Legume**	**Protease Target**	**Fungus Pest Target**	**Molecular Mass (kDa)**	**N-terminal Amino Acid Sequence**	**Reference**
*Psoralea corylifolia*	Trypsin	*Alternaria brassicae, Aspergillus niger, Fusarium oxyxporum, Rhizoctonia cerealis*	18	EWEPVQNGGSSYYMVPRIWA	[54]
*Acacia plumosa*	Trypsin and chymotrypsin	*Aspergillus niger, Thielaviopsis paradoxa, Colletotrichum* sp.	20	KELLVDNEGEI	[55]
Chickpea (*Cicer arietinum*)	Trypsin	*Fusarium oxysporum*	20	ND	[56]
Lupin (*Lupinus albus)*	Cysteine protease	*Fusarium oxysporum, Botrytis cinerea, Alternaria solani,* *Aspergillus niger, Penicillium expansum*	10.7–11.8	ND	[57]
Peanut (*Arachis hypogaea*)	Trypsin and chymotrypsin	*Aspergillus parasiticus*	16.82	ND	[58]
Black soybean (*Glycine max L. merr*)	Trypsin and chymotrypsin	*Alternaria alternata, Fusarium oxysporum, Pythium aphanidermatum, Physalospora piricola, Botrytis cinereal, Fusarium solani*	20	DEYSKPCCDLCMCTRRCPPQ	[59]
Mung bean (*Phaseolus mungo*)	Trypsin and chymotrypsin	*Physalospora piricola, Mycosphaerella arachidicola, Botrytis cinerea, Pythium aphanidermatum, Sclerotium rolfsii, and Fusarium oxysporum*	10	EMPGKPACLDTDDFCYKP	[60]

ND. Not determined or not reported.

## Abbreviations

ABA	Abscisic acid
ANFs	Anti-nutritional factors
ET	Ethylene
ETI	Effector-triggered immunity
ETS	Effector-triggered susceptibility
HGPs	*H. armigera* gut trypsin-like protease
HR	Hypersensitive cell death
JA	Jasmonic acid
NB-LRR	Nucleotide-binding and leucine-rich repeat domains
PA	Phosphatidic acid
PAMPs	Pathogen-associated molecular patterns
PIs	Protease inhibitors
PR	Pathogenesis-related
PRRs	Pathogen recognition receptor
PTI	PAMP-triggered immunity
SA	Salicylic acid
